# Referring psychiatric patients to occupational health services for earlier return to work – a qualitative implementation study of barriers and facilitators

**DOI:** 10.1186/s12913-025-12238-2

**Published:** 2025-01-20

**Authors:** Mikko Henriksson, Christina Tikka, Pirjo Juvonen-Posti, Marianna Virtanen, Tuula Oksanen

**Affiliations:** 1 Finnish Institute of Occupational Health, Unit of Work Ability and Working Careers, Topeliuksenkatu 41b, Työterveyslaitos, P.O. Box 40, Helsinki, 00032 Finland; 2 University of Eastern Finland, School of Medicine, Institute of Public Health and Clinical Nutrition, Yliopistonranta 1, Kuopio, 70210 Finland; 3 University of Eastern Finland, School of Educational Sciences and Psychology, Yliopistokatu 2, 80100 Joensuu, P.O. Box 111, Joensuu, 80101 Finland

**Keywords:** Mental health disorders, Work disability, Sickness absence, Implementation research, CFIR, RTW, Cooperation between health care providers, Digital/electronic referral, Occupational health services (OHS), Psychiatric care

## Abstract

**Background:**

Mental disorders are a major public health challenge, and their prevalence is globally increasing. They substantially affect work ability, quality of life, and the number of years of disability. A new model for referring psychiatric patients to occupational health services (OHS) aims to improve the continuity of care and to promote the early return to work (RTW) of workers with diagnosed mental health conditions. The purpose of this qualitative implementation study was to identify the facilitators of and barriers to implementing the new model.

**Methods:**

We used the Quality Implementation Framework and the Consolidated Framework for Implementation Research (CFIR) as theoretical frameworks. We interviewed the developers of the model and the psychiatrists and occupational health physicians who deliver it. We invited forty participants to join the study, 17 of whom consented. We conducted nine semi-structured group and individual interviews. Data analysis consisted of analysing the sessions, systematically coding the transcribed texts according to the main domains of CFIR, thematic analysis, and identifying the overarching themes and context-related mechanisms.

**Results:**

We identified three overarching themes crucially related to the implementation of the model: uncertainty about the scope and boundaries of the cooperation in the model, ambiguity about the size of the target group, and the existing sociocultural and self-stigma related to mental illness. Shared belief in the importance and the positive effects of the model and trust in the developers were the main facilitators of the implementation of the model. The main barriers were the limited availability of the e-referral system between the psychiatrists and OHS, uncertainty regarding the number of eligible patients, and the low number of actual referrals during implementation.

**Conclusion:**

Collaborative models in mental health care should accommodate various stakeholders from different sectors involved in the treatment and rehabilitation of workers with diagnosed mental health conditions. Helping health care workers contact possible cooperation partners and knowing how to address important individual, workplace-related and sociocultural factors such as stigma may strengthen collaboration between different sectors and stakeholders in mental health care. Future studies should focus on the multi-actor feasibility of the new collaborative models and include the patients’ perspective.

**Supplementary Information:**

The online version contains supplementary material available at 10.1186/s12913-025-12238-2.

## Background

Mental disorders are a major and increasing public health challenge, constituting a substantial global burden in terms of years of disability [1]. They are also the leading cause of sickness absence and work disability pension in most high-income countries, including Finland, where mental disorders have been at the top of compensated sickness absence benefits since 2018 [2]. The authors of the Global Burden of Disease Collaboration concluded that the global community needs coordinated prevention strategies and treatment programmes to reduce the burden of mental disorders [1]. The gap between required treatment and the treatment received must be reduced, as help-seeking in cases of mental disorder is rare, and the treatment received by those seeking help is insufficient. In addition to the gap between required and received treatment, there are also issues in the quality of care.

According to the European Psychiatric Association, clinical care should include evidence-based diagnostic and treatment procedures, such as the use of screening instruments and tests to detect common mental disorders; and psychiatric care, such as psychotherapy, pharmacotherapy, and initial psychiatrist appointments should be more easily available to people with depression [3–5]. Further, treatment should incorporate a holistic approach that accounts for all relevant life areas, including working life. This requires stigma-free contact between the employee and employer to enable the changes in the workplace that are needed to support the employee’s return to work (RTW) [3]. This is in line with evidence from previous reviews [6–8]. The combination of clinical and workplace support interventions effectively reduced sick leaves among employees with common mental disorders, such as depression and anxiety disorders, but psychological or workplace interventions alone did not have the same effect [6–8].

However, in many countries, mental health care is fragmented and unequally distributed. In Finland for example, shared care and service chains (care pathways) either do not exist or are incomplete, the service structure is fragmented, waiting lists for specialised care are long, and the body responsible for the patient’s treatment is not always clear [4, 5, 9]. In Finland, according to the Occupational Health Care Act [10], employers must organise occupational health services (OHS) for all employees to protect and promote their work ability, including measures needed to support RTW. However, as the level of clinical treatment depends on the employer’s willingness to cover the costs, it varies between workplaces [11]. Regarding specialised mental health care, such as psychiatric consultations, these may be provided in public specialised health care, primary health care and OHS. Inpatient mental health care is provided in public hospitals only.

Inspired by chronic disease models in somatic health care settings [12], collaborative care models have been developed to improve treatment for mental disorders. A collaborative care model can be defined as intensified, coordinated collaboration between primary or occupational and specialised mental health care providers. However, collaborative care can be difficult to implement because it is a complex intervention consisting of several changing components and requires commitment from several parties, including the patients themselves and primary or occupational and specialised health care. Therefore, it is important to identify the facilitators of and barriers to successful implementation, which was the topic of a qualitative systematic review focusing on primary mental health care [13]. Findings from studies in North America and the UK have revealed that resource allocation, education, the face-to-face availability of a case manager and the quality of communication systems were the most important facilitators of and/or barriers to implementation [13]. An intervention study in Norway transferred mental health professionals from specialised mental health services to primary health care centres and identified common locations and the availability of low-threshold mental health specialisation as facilitators, and the greatest barrier to collaborative care was the lack of financial support to fully implement the model [14].

In Sweden, the facilitators and barriers were identified in the implementation of the coordination of RTW between different actors when focusing on primary mental health care [15]. The study authors found that positive attitudes, open dialogue, leadership engagement, routines, structures for RTW practices, and collegial alliances facilitated implementation. The barriers included insufficient communication about the intervention, conflicts at the workplace, lack of team-based work and varying competence levels among the coordinators.

Few studies have analysed the implementation processes of collaborative care from the perspective of specialised mental health care settings. In Canada, the implementation of an RTW programme with occupational therapists as coordinators was found to support the RTW of people with common mental disorders [16]. In Australia, the transition from specialised mental health care to primary care for individuals with comorbid mental and physical diseases was facilitated by the clinicians’ expertise, engagement in the project and accessibility (care pathways), whereas few or no care pathways, poor communication, poor continuation of care and negative attitudes were barriers to a smooth transition [17]. Clear and structured care pathways can provide patients with a clear picture of their future care and improve teamwork, the use of guidelines, and the measurement of patients’ progress [18]. Successful implementation of care pathways can be facilitated by, for example, better clinical and management staff involvement, flexibility, context specificity and financial incentives. The long list of barriers included time constraints, lack of resources, lack of knowledge and training on the intervention and the rigidity of the implementation [18]. Negative attitudes among the health care personnel may reflect mental health-related stigma, which exists from the individual (patient) level to organisational and structural levels, and which has been identified as a major barrier to recovery from mental disorders [19].

In Finland, improving collaboration between specialised mental health care and OHS and strengthening care pathways to improve the continuation of care and RTW are seen as important developmental targets to prevent long-term work disability in the working population [20, 21]. A new referral model based on an earlier model that focused on musculoskeletal diseases [22, 23] was expected to reduce the duration of long-term sick leaves by enabling quicker RTW of employees. It was also expected to reduce the workload of psychiatrists in special care by enabling work ability assessments and the related preparation of certificates to be transferred to OHS. Patients’ employers were expected to benefit by raising the number of employees returning to work earlier, and freeing up public resources for those who have no access to OHS [24, 25]. This new referral model was implemented straight after the COVID-19 pandemic as it was hoped it would create partial solutions to the mental health effects of the pandemic on working life. At the same time, Finland was undergoing a public health care reform that would transfer the responsibility of organising health care, social welfare and rescue services from municipalities and joint municipal authorities to well-being services counties.

When developing collaboration between mental health care and vocational rehabilitation to support RTW, better understanding of the facilitators of and barriers to implementation is needed. To the best of our knowledge, no previous studies have evaluated the implementation of referral models involving different health care sectors in mental health. The aim of our study was to investigate these facilitators of and barriers to the implementation of a new referral model for mental health patients from specialised mental health care to the OHS in order to increase earlier RTW.

## Methods

### Study design

This qualitative implementation study examined the facilitators of and barriers to a new referral model that was implemented in two well-being services counties in Finland from 2021 to 2023. We utilised the Quality Implementation Framework (QIF) [26] to construct the interview themes. We employed a data collection process informed by grounded theory [27, 28]. The CFIR [29] was used as the theoretical framework for the deductive analysis of the interview data. We also conducted inductive thematic analysis and identified the context-related mechanisms. The data were analysed independently by three of the authors and consensus was reached through discussion.

We collected data from the viewpoints of the developers of the referral model and the deliverers of the services (psychiatrists and occupational health physicians) because the development and implementation was based on multi-actor cooperation [30, 31]. The patients were not the focus of this study as the model was specifically designed to focus on enhancing professional collaboration.

### Enrolment and interviews of participants

Our study focused on the perspectives of those involved in the development and implementation of the referral model, that is, the developers, psychiatrists and occupational health physicians. We used qualitative methods to elicit the perspectives and experiences of those who were central to the development and implementation of the referral model [32]. We aimed to carry out six group interviews: one group interview with the developers of the new referral model and five group interviews with psychiatrists and occupational health physicians from two well-being services counties [33–35]. The developers provided us with a list of 40 email addresses (including the developers themselves) of individuals who had at some point participated in developing or implementing the new model. The listed potential participants were distributed evenly within each of the two participating well-being services counties. They were contacted via email; and 17 gave their informed consent to participate. Most of the participants (14/17) were psychiatrists or occupational health physicians; the remaining three participants were health care professionals also responsible, in various ways, for the implementation of the referral system.

Due to scheduling issues, a total of nine interviews were held, comprising five group interviews and four individual interviews. Two researchers jointly conducted all the interviews between March and May 2023, using semi-structured interview guidelines (Supplement 3.) and taking interview notes. The interviews were conducted remotely via telecommunication software (Microsoft TEAMS) and recorded on an external audio source for pseudonymised text transcription. A total of 461 min of recorded conversation were transcribed into pseudonymised transcriptions, resulting in a dataset of 186 pages. The purpose of the sixth group interview was to explore potential challenges related to the model's use by a group that had not yet started collaborating in accordance with it. After conducting five interviews, we took a reflexive stance, which lead us to scrutinize our data [36] and found that we had gathered substantial information about the barriers to implementing the referral model. Based on the principles of grounded theory-informed data collection, we decided not to conduct the sixth group interview as originally planned. The developers of the referral model were interviewed first, and then the psychiatrists and occupational health physicians.

The QIF was applied to guide the development of the interview themes [26]. This ensured that the interviews captured all the important aspects of the dynamic and evolving implementation process. Interview themes focused on the barriers, facilitators, and development ideas associated with the preparation and actual implementation process as well as the context and possible impact of the referral model on RTW ([26], Supplement 3.). It was important to use context-specific interview themes, because contracts between OHS providers and workplaces can vary. Differences in contexts may impact interview participants’ perceptions of the effect of the model on RTW and other outcomes [37, 38].

### Description of the analysis process

The analysis process consisted of 1) sessions after the interviews analysing each perspective in each regional context, for example, after interviewing the occupational health physicians of one well-being services county, 2) systematic deductive coding of the transcribed texts according to the main domains of the CFIR, 3) thematic analysis to inductively derive themes from the data [39, 40], 4) identifying the overarching themes that crossed the CFIR domains [41], and 5) identifying the dynamic mechanisms based on the identified facilitators of and barriers to implementation [42].

Five analysis sessions were held during the data collection phase, i.e., a joint analysis session of two researchers, who gathered the interview data as soon as all the interviews from the selected perspective had been carried out: one after interviewing the developers and one after each group of interviews of psychiatrists and occupational health physicians from a certain area. These two researchers identified statements and initial patterns that they deemed relevant to the research questions and reviewed the interview themes for the next interviews.

Two researchers conducted systematic classification by coding the data into implementation facilitators and barriers under the five main updated CFIR domains: innovation characteristics, inner setting, outer setting, characteristics of the individuals involved, and the implementation process [29]. Due to the relatively small amount of interview material, we did not utilise the CFIR constructs under the domains. We operationalised the CFIR prior to the data collection by defining the subjects of the domains into a project-specific level as follows: the new referral model under investigation (innovation domain), the actors in the development project and those implementing the model (inner setting domain), the operating environment (outer setting domain), and everything related to the implementation process itself (implementation process domain). The individual domain was divided into 1) psychiatrists’ and 2) OHS personnel’s points of view, and 3) psychiatrists’ and OHS personnel’s perspectives on the patient’s viewpoint. The latter distinction was made because even though we were not able to gather data directly from the patients, psychiatrists and OSH personnel provided second-hand and hypothetical data about patients’ viewpoints. Coding used software for qualitative data analysis (Atlas.ti programme) [43].

Following the systematic classification, we employed thematic analysis to identify the themes and phenomena from the quotations coded into different CFIR domains [39, 40, 44]. First, three researchers equally divided the data set and independently developed initial themes. They then continued collaboratively developing and further finalising the themes until a sufficient interpretative consensus was reached [39, 40, 44]. Several discussions were held via Microsoft TEAMS during the analysis and reporting process.

By embedding the CFIR and thematic analyses and quantifying measurable aspects [45]— specifically, discerning the frequency of the most often mentioned phenomena within the final themes — three overarching themes were formulated and defined [41]. Altogether, these themes represent not only the most frequently cited aspects across different domains but also those deemed crucial to address on the basis of the researchers' expertise in the pertinent themes. Subsequently, the analysis extended from examining certain facilitating factors or barriers to identifying context-related mechanisms [46, 47] that influenced the promotion or hinderance of the implementation of the referral model. These mechanisms emerged as distinct phenomena articulated by the interviewees.

## Results

We summarised our results regarding the facilitators to and barriers of the new referral model into three overarching themes and six context-related mechanisms which reflected broader phenomena influencing the implementation process. Next, we describe the overarching themes.

### Overarching themes

During the analysis, we discovered and formed three overarching themes A, B and C. Theme A – ‘Discussion of the scope and boundaries of the cooperation in the new referral model: What was the innovation?’ – emerged in the data in all the CFIR domains as both a facilitator and barrier, except for in the Innovation domain and the Implementation Process domains, where it emerged as only a facilitator or barrier respectively (Supplementary Table 1). Theme B – ‘Size of target group smaller than expected’ – mainly emerged in all the main domains as a barrier, and Theme C – ‘Importance of stigma associated with mental health problems in the return-to-work process’ – in all but in the Inner Setting domain as a facilitator or barrier (Supplementary Table 1). All these overarching themes were particularly important for successful implementation of the model.

A. Discussion of the scope and boundaries of the cooperation in the new referral model: What was the innovation?

The narrowest definition of the implemented innovation was the e-referral from the psychiatric clinic to the OHS provider. However, the interviewees described the cooperation not only according to the model but in diverse ways, either broadly or precisely. Some considered other health care services such as primary health care to be part of the new model. They also considered cooperation with employers and other workplace actors to be part of the innovation. Implementation was clearly weakened if the actors had no common understanding of the scope and boundaries of the innovation. Even if the scope and boundaries had been explained more efficiently, the service deliverers might still have had difficulties focusing solely on the one-way aspect of collaboration from psychiatrists to OHS, given the many sectors involved in mental health care.

B. Size of target group smaller than expected

According to the referral model, psychiatric professionals of working-age patients are advised to discuss working life issues and work ability support needs with the patient, and to obtain permission to transfer the patients’ clinical data to the OHS provider. Some interviewees estimated that thousands of employed persons in psychiatric outpatient care could annually benefit from the new model in the two well-being services counties. However, during the implementation, there were only a few or no referrals of such patients to the OHS, depending on the area in question. Further, the number of eligible patients was unclear to the deliverers. Together, these formed a major barrier to the successful implementation of the referral model.

C. Importance of stigma associated with mental health problems in the return-to-work process

The stigma associated with mental illness has negative consequences for all aspects of life for those who encounter it. The interviewees reported issues with stigma related to mental health disorders in all the stages of implementing the referral model, from seeking treatment to returning to work and the workplace. Stigma was described as both a sociocultural phenomenon, such as attitudes within OHS or workplaces, and as self-stigma.

### Context-related mechanisms as phenomena

After identifying the individual facilitators of and barriers to implementation in the CFIR domains, we continued the thematic analysis to explore the dynamic mechanisms that the individual factors formed and that could either hinder or speed up implementation (Supplementary Table 2). These mechanisms were articulated by the interviewees and are presented as the following six phenomena.

- Developers and health care providers share the same belief that the innovation has an important and positive effect

The deliverers of the innovation supported its core idea and implementation. The developers and deliverers were both motivated by the shared belief that supporting workers with mental health issues is important and that the innovation would have a positive effect on patients’ health outcomes, quality of life and RTW. The private OHS professionals were ready to intensify cooperation with the psychiatric public health care sector as part of the innovation. However, some key actors did not understand the work ability issues in the same way as the innovation required.

*“The individuals using the services, if we now refer to them as [...] patients, will definitely benefit [...] unnecessary long sick leaves, for example, could be avoided. [...] returning to work could then be possible earlier, for instance.” Interview 3, psychiatrist*

*“Well, if we consider how mental health disorders lead to early retirement, the retirement among those under 35 is absolutely significant. And considering how much mental health issues contribute to work disability and retirement, it's, of course, crucial to receive assistance and support, especially from occupational health services, where they have a more detailed understanding of the employee's job description, work-related stressors, both mental and physical aspects.” Interview 4, psychiatrist*

b. Need for clear roles and responsibilities for all involved in the treatment and rehabilitation process of workers with mental health problems – not only OHS and psychiatrists

There were some ambiguities about roles, resources and the nature of the cooperation needed to implement the innovation. The focus of the model did not include all the cooperation partners and treatment pathways needed to answer the challenges related to mental health and RTW. Instead, the innovation focused solely on the one-way referral path from specialised public health care (here, psychiatry) to OHS. This was a barrier to implementing the new model in two senses. First, the collaborative line from psychiatric public health care to OHS can only be separated for analytical purposes from the collaborative sphere of mental health care actors in primary health care, OHS, and specialised mental health care. The collaboration should be developed as a whole, i.e., including public, private, and non-governmental health care services and the social insurance institution. Also, all treatment paths and collaboration lines should be comprehensively examined. Second, the sphere of collaboration was so essential to the actors themselves that it was sometimes difficult to keep the interviewees on the topic of developing collaboration between the psychiatrist and the occupational health physician, and they often started talking about all the actors involved.

*"And to some extent, on the psychiatric side, there was a bit of getting used to, you know [...], wondering if there would be some back and forth or [...] questioning whether the statement from the occupational health physician is sufficient for the pension institutions or if it still needs to be the psychiatrist's statement. And there was some [...] feedback that, after all[...], [Finnish Social Insurance Institute] had demanded a psychiatrist's statement and so on." Interview 3, psychiatrist*

Supporting RTW and continuing psychiatric treatment simultaneously demands proactive and circumstantial collaboration and the ability to understand the mechanisms of this process. The psychiatrists' motivation for collaboration with OHS was twofold. They saw an opportunity to ease their own intense workload by shifting the responsibility of writing medical certificates to OHS as well as to improve the RTW support and quality of life of the patients.

*"...we could shift that [psychiatrists'] pressure of providing statements away from us, and also, since there is genuine expertise there [in occupational health services], in how to support the return to work [... ]psychiatrists, on the other hand, are increasingly becoming fewer, or rather, a disappearing resource, so, it's going to be difficult in any case." Interview 2, psychiatrist*

As a contextual factor, there were ambiguities and different expectations in the practice of defining the need for sick leave. The psychiatrists knew that according to the model, OHS should have prescribed sick leaves. However, during the implementation, the psychiatrists may have needed to define sick leave in some cases during the patient’s first appointment, even though the consulting occupational health physician would have been more familiar with their work and working conditions, making them better able to assess and prescribe the need for sick leave.

*“There might still be work to be done[...] We probably wish on the side of occupational health services that sick leaves would be recommended, not necessarily by psychiatrists suggesting a specific timeframe but leaving [....] the assessment of work ability to the field of occupational health.” Interview 7, occupational physician*

*“[…] there are situations [...], where if someone is completely incapacitated and can't even get out of bed for the entire day [...], a psychiatrist would probably write those sick leaves.” Interview 3, psychiatrist*

c. Need to ensure functionality of all innovation components and correct timing of the implementation

The unavailability of e-referrals was one of the most significant practical implementation barriers of the new model. E-referrals should have been a crucial part of the new referral model. However, the development of the ICT system was an ongoing process, and it took time for the system to become operational. When available, the psychiatrists felt that the e-referrals were easy to use.

*“...this (hasn't been) so much from the psychiatric side, but rather more from the hospital information management and then these information technology companies or agencies that handle these information system changes for the hospital districts, so working with them has taken a really long time.” Interview 5, developer*

Furthermore, the deliverers of the innovation perceived that the timing was not optimal. The ongoing public health care reform was causing nationwide regional structural and organisational changes. The restructuring of national social and health care began in Finland on January 1, 2023. Preparing and establishing the new ‘well-being services counties’ at the same time as implementing the innovation weakened the commitment and practical opportunities related to the new e-referral model.

*“And probably one slowing factor there has been that simultaneously, there has been a change in the well-being area organization [long pause] and that has, of course, been a significant effort also on the information management side, so they now have such a high workload and congestion related to these organizational changes that they may not have found so much time for these smaller change*s.” *Interview 5, developer*

The personnel shortage in the health care services, especially of psychiatrists in the public health care sector, was a common problem. Thus, some of the deliverers reported difficulties developing and implementing the projects.

“.*..practical challenges probably had an impact, such as the shortage of doctors, at least now in the psychiatric field. In occupational health, the situation might be slightly better. And there are rotating doctors, those who work for short periods[...] Well, those things always affect the functionality of collaboration with partners like this.” Interview 3, psychiatrist*

d. Ambivalence towards OHS competencies and resources in supporting the return to work of employees with mental health problems

The OHS providers reported a readiness to cooperate with psychiatric public health care services in the way described in the innovation. The roles and responsibilities within OHS were clear in cases of referrals by a psychiatrist. However, it was unclear to the psychiatrists and occupational health physicians whether OHS had adequate competencies and resources for dealing with mental health problems. Although they had confidence in the OHS regarding work ability assessments and practices for initiating and following up on the RTW process, some claimed that OHS professionals’ knowledge and competencies for care for patients with mental health problems needed improvement. This seemed especially important, as some health care providers expected an increase in the number of workers with mental health problems and thus an increase in their workload with these patients in the future.

*“...we rely on the fact that occupational health has the good assessment tools and connections to the workplace so that all possible support for returning to work and restoring or maintaining work capacity can be utilized to support people's recovery and work ability.” Interview 3, psychiatrist*

*“...probably the need for that is quite strong in terms of work ability management, and as these [mental disorder category]-diagnoses increase, this expertise must indeed be acquired, probably a bit everywhere, in fact. [...] we have at least made the decision to internally train occupational health nurses in short-term therapy-related expertise and probably this kind of interaction, which is then a bit of a shorter training.” Interview 10, occupational physician*

*“And then we consider that if there's a longer sick leave, then typically, either an occupational health negotiation or at least a visit to the doctor here, possibly during the return-to-work phase. So, we make good use of that part-time sick leave during the return to work if it's a longer sick leave, and it's easier to arrange from here.” Interview 8, occupational physician*

e. Individual, workplace related and societal factors that can affect the return-to-work process were not addressed by the innovation

From the interviews with the psychiatric and OHS providers it became clear that some important factors related to RTW were not addressed by the new referral model. Factors that should have been considered were the attitudes and beliefs related to mental health problems. Stigma in the workplace as well as self-stigma were described as barriers to patients seeking treatment, to permitting the transfer of data from psychiatric care to OHS, and to RTW. On the other hand, accepting attitudes and positive beliefs of different actors could support and facilitate treatment-seeking, cooperation, and RTW. In addition, it was highlighted that work accommodations depended on the type of work and on the employers’ and supervisors’ readiness to modify the job to the person’s work ability. Thus, RTW to the same workplace might not always be possible. The health care providers expressed these as limitations to the innovation and as barriers to its implementation.

*“ ...maybe more specifically the challenges on the psychiatric side are wanted to be sort of ruled out [at workplaces]. So, this is how I see it a bit, maybe some kind of taboo still.” Interview 8, occupational physician*

“*...some of these recovering individuals may suffer from intense feelings of shame, for example, and from the experience of failure, inadequacy, and disappointment with themselves for having to take time off work due to this type of illness. It would be easier if they had a broken leg [...] to just say that this is the situation. [...] And that's why I then think about how important it is [...] that a supervisor has a positive attitude towards this type of employee when returning to work and perhaps an accepting attitude. And specifically, the opportunity to arrange work conditions in a way that provides flexibility, the possibility of remote work, and other aspects related to working hours.* “*Interview 3, psychiatrist*

f. Difficulties estimating the size of the target group

Even though it was estimated that there were possibly thousands of potential joint customers every year, the number of employees eligible for referral from psychiatrists to OHS was unclear to all involved in the interviews, including the developers of the innovation.

*“I haven't received a systematic report so that I would know exactly how many referrals we've had. But I would imagine that we do get referrals weekly.” Interview 7, occupational physician*

*“Especially in the early stages of the project, there was quite a bit of discussion about how much of the patient population this issue even applies to—given that over a third, [pause] or even more, of the patients are under 22 years old and definitely do not have any occupational health care. Then there's a portion of the patients who do not have jobs [pause] considering how much of that ultimately involves dealing with this issue, collaborating and contemplating with occupational health services. And is it possible [...] to consider a return to work in such cases...” Interview 2, psychiatrist*

The health care providers highlighted that although various treatment paths for patients with mental health problems existed, the innovation focused solely on psychiatrists' referrals to OHS. Therefore, not all employees with mental health problems seeking or currently receiving psychiatric care were reached by the innovation.

## Discussion

### Summary of the main findings

The three overarching themes affecting the implementation of the new model for referring mental health patients from specialised mental health care to OHS for earlier return to work were A) discussion of the scope and boundaries of the cooperation in the new referral model: What was the innovation? B) size of target group smaller than expected, and C) the importance of stigma associated with mental health problems in the return-to-work process. Rather than examining individual barriers and facilitators in isolation, we also identified six dynamic phenomena that emerged from these factors, each capable of either hindering or facilitating the implementation process, depending on the context. These phenomena were constructed from the observed facilitators and barriers and reflected broader mechanisms that shifted according to situational factors. There was support for and belief in the effect of the new referral model (Phenomenon a), trust in the developers (Phenomenon b), and trust in the skills of the OHS personnel to assess and support the RTW process (Phenomenon d). However, the required ICT systems were not readily available at the start of the project (Phenomenon c). Further, the health care providers and developers were uncertain about how many employees with mental health issues could be reached by the new model (Phenomenon f). Some of the main uncertainties concerned the adequacy of the competencies of the OHS to address the RTW issues of workers with mental health issues (Phenomenon d), the feasibility of the new model for all RTW processes, and whether the new referral model would address the stigma related to mental health (Phenomenon e).

Furthermore, the health care providers encountered challenges in implementing the innovation due to the general shortage or turnover of staff and the newly initiated public health care reform. Overall, there was uncertainty regarding the number of patients eligible for a referral from public psychiatric care to OHS (Phenomenon f).

### Comparison with other studies

Strengthening the collaboration between occupational and public health stakeholders is increasingly being seen as an important intervention to prevent work disability and long-term sick leave in the working population. Communication between the sectors is seen as especially important when addressing shared health hazards or health conditions, such as during the COVID-19 pandemic [48] or when addressing work rehabilitation [49, 50]. In our study also, the psychiatrists considered the collaboration defined by the new referral model as important because OHS have the connections and knowledge regarding the work and workplaces, and the psychiatrists could not and would not want to take part in the work ability assessment process without knowing the actual work and workplace contexts.

Conceptualisations of work ability and disability have evolved significantly and become more holistic in recent decades [51]. Systematic frameworks, such as those described by Lederer et al. [52], address the various factors that influence work ability. This marks a shift away from the traditional biomedical models commonly used in social insurance contexts, which explain disability solely in terms of illness. The newer perspective views work ability and disability as dynamic, multilevel concepts, incorporating social, organisational, and societal factors [52]. Our interviews on psychiatric care also acknowledged this shift. Although the psychiatrists admitted that they lacked the data and tools to directly assess work ability, they acknowledged the importance of understanding its systemic nature. At the same time, they stressed the need for individualised work ability assessments in social insurance and workplace contexts.

Our study highlighted the significance of external factors. For example, the unavailability of the e-referral system was the primary barrier to implementation. Similarly, the feasibility of the Therapeutic Return-to-Work Program in Canada in a specialised mental health hospital was found to be highly promising for supporting the RTW of workers with common mental disorders. However, it was emphasised that the facilitating contextual factors would need to be identified before large-scale implementation [16]. In line with these observations, the British Medical Council’s updated framework for developing and evaluating complex interventions underscores the critical importance of thoroughly considering contextual factors during the implementation process before moving on to assessing an innovation’s effectiveness [46].

Previous studies in Finland, Sweden, and the Netherlands have identified patients’ and health care providers’ experiences, expectations, and needs regarding coordinating occupational and other health care services, with similar results to our study. In Finland, increasing communication and coordination between public and occupational health services was perceived as important by health care professionals from both sectors but was considered difficult to establish [50]. In Sweden, the implementation of a similar intervention to enhance communication and coordination between the public and occupational health services was analysed [15]. Although the design of the innovation was more detailed than the model evaluated in this study, the results also showed that its implementation would have benefited from a more accurate description of the boundaries of the model. In the Netherlands, the patients’ experiences, needs and expectations regarding cooperation between medical specialists and occupational health physicians were identified. Although we did not include interview data from patients, the results of the study align with the findings of our study, showing that patients’ job retention and RTW outcomes seemed to benefit from improved cooperation [53].

### Implications for practice and research

Workplace modifications, such as working part-time or not doing night shifts, can enhance sustainable RTW [6, 54] and OHS interventions that encompass both clinical and workplace support can effectively facilitate the RTW of employees with common mental disorders [6, 55]. Similarly, the new referral model aimed to ensure that the treatment and support from psychiatric and occupational health services complement each other and can be administered simultaneously. This should also be included in future development projects.

Further knowledge is needed of how this innovation co-exists or interacts with other available innovations in Finland, such as the Mental Health at Work Programme, which focuses on the cooperation between workplaces and OHS [56].

In the future, further resources may be needed to increase the number of patients agreeing to and psychiatrists issuing a referral to OHS. This objective may be achieved by initially evaluating the competency levels of OHS professionals concerning mental health-related issues and enhancing these competencies through targeted training. This training should encompass not only common mental health conditions but also treatment methodologies and workplace accommodations. Furthermore, it is essential to enhance psychiatrists' understanding of the skills and competencies of OHS professionals in the context of mental health and return-to-work (RTW) processes. Additionally, it is critical to inform employees and employers about potential workplace modifications to support RTW and mental health. Further, more guidance for health care providers on how to address patients’ concerns and share their mental health data with OHS, and how to address issues related to mental health stigma at workplaces may positively influence the collaboration between patients, employers, mental health care, and OHS providers. Both educational strategies and enhancing interpersonal contacts should be considered in addressing the issue of stigma.

Also, if psychiatrists were better informed or reminded about when patients should be referred to OHS, cooperation between OHS and psychiatrists, as well as the number of referrals, may increase. At present, although psychiatrists are provided with a list of referral indicators and examples, they need to remember when to discuss the topic with the patient and when to initiate the referral. Inclusion of the new referral model in regional integrated care pathways or the national Current Care Guidelines may further improve the identification of eligible patients and increase the number of referrals.

The aim of the innovation under study was to reduce the duration of sick leaves issued by psychiatric doctors and to increase patients’ RTW. Our study showed that health care workers believed that the new model would positively affect patients’ RTW and quality-of-life outcomes. However, the number of psychiatric patients eligible for referral to OHS was not clear to all involved. The results also showed that a better estimation of the size of the target group is needed during the pre-implementation phase. Future studies may also need to evaluate how the new referral model between psychiatrists and OHS influences physicians’ sick leave prescription practices.

In our study, the psychiatrists reported a lack of human resources to implement the innovation. The cost of implementing these types of innovations should be assessed thoroughly, as should the resources needed for collaboration and setting up ICT services between different health care sectors. More importantly, our results indicate that in the future, instead of focusing on one specific referral pathway only, such as that from psychiatrists to OHS, all mental health care providers may need to be included in efforts to improve the RTW of workers with mental health problems.

### Strengths and limitations of the study

Our study is the first to analyse the implementation of the new referral system for employees with mental health problems in Finland. We discovered three overarching themes and six phenomena that were particularly important for its successful implementation. The analysis process shifted from deductive, linked to the metatheoretic framework, to inductive, and required a great deal of time. This turned out to be a strength in finalising the themes and results of the multi-scientist collaboration, which crossed both organisational and scientific field boundaries. We believe that lifting the analysis from the CFIR domains to identifying overarching themes and phenomena enriched the study significantly.

Although only 17 out of the 40 invited individuals participated in the interviews, our sample included almost half of the primary actors responsible for developing and implementing the model. The results from our interviews thus provide a valid insight into the perspectives of both developers and deliverers. Although more barriers or facilitators may have been identified if we had interviewed more health care providers, our results still show a wide range of themes that were repeatedly identified in the interviews. Thus, it is likely that we identified the main facilitators of and barriers to the implementation of the model that OHS providers and psychiatrists in Finland are facing.

Further, the participants' willingness to enrol in the study and discuss various topics may have been influenced by the interview methods used. Using both group and individual interviews ensured that we reached all the enrolled participants. Even though our study only had a small sample, OHS and psychiatric services are very similarly organised throughout Finland, and thus our findings can be applied to other regions and well-being services counties in Finland. The application of our study findings to countries other than Finland may only be possible to a limited extent, as health care services and systems differ between countries, especially outside of Europe. However, mental health issues are on the rise globally, making the relationship between OHS and psychiatric care increasingly important to enable these challenges in workplaces to be managed.

Our study focused on the perspectives of those involved in the development and implementation of the innovation, i.e., developers, psychiatrists, and occupational health physicians. Additional perspectives, such as those of patients, employees, or ICT system experts, would have further strengthened our study results. However, we were unable to include additional perspectives as we lacked the necessary resources. The focus of the new referral model was to improve the collaboration of professionals. However, workers’ and employers’ perspectives of the innovation might have helped clarify the stigma related to mental health and RTW, the timing of the RTW process, and the roles and responsibilities of OHS and workplace representatives in supporting mental health [21].

## Conclusions

Our study provides valuable lessons for decision-makers on how to improve the cooperation between specialised mental health care and OHS. Health care providers’ shared belief in the relevance, importance and positive effect of the innovation and their trust in the developers were important facilitators of the new referral model. However, the limited availability of the e-referral system between the psychiatrists and OHS, the ambiguity regarding the size of the target group, the innovation not addressing mental health stigma, and the lack of clarity about the innovation itself hindered implementation.

The barriers to the implementation of the innovation could be overcome by two factors. First, better communication of the context of the referral model. Second, better guidance or tools for health care professionals for a) identifying eligible patients, b) contacting possible cooperation partners, and c) addressing important individual, workplace-related and sociocultural factors such as stigma.

Beyond further developing and implementing the model, our findings can contribute more broadly to enhancing the quality of care, vocational rehabilitation, and RTW support for employees with mental disorders. First, the collaboration among the various sectors and stakeholders in mental health care should be improved comprehensively. This will require resources to enhance OHS professionals' understanding of mental health and psychiatrists' knowledge of the RTW process. Second, methods for evaluating the size of the target group need to be improved, such as developing and integrating patient tracking systems across various service providers to more accurately estimate and monitor the number of eligible patients. Development of collaborative mental health care also requires considering the factors in the operational environment, including workplace heterogeneity and issues related to stigma.

## Supplementary Information

Supplementary Material 1.

## Data Availability

The qualitative interview material generated and analyzed during the current study is not publicly available to maintain the anonymity of participants. However, the authors have anonymized the material and citations before submitting the manuscript. Requests for access to the anonymized data can be directed to Mikko Henriksson/Finnish Institute of Occupational Health upon reasonable request, taking into consideration the need to uphold participant confidentiality.

## Abbreviations

CFIR: Consolidated framework for implementation research
ICT: Information and communication technology
N: Number
OHS: Occupational health services
PS: Psychiatrists
RTW: Return to work
