# Supplementary material for: Referring psychiatric patients to occupational health services for earlier return to work – a qualitative implementation study of barriers and facilitators
Source: BMC Health Serv Res. 2025 Jan 20;25:109. doi: 10.1186/s12913-025-12238-2 (PMC11748852; doi:10.1186/s12913-025-12238-2)
Supplement: Supplementary file 1 — Supplementary Material 1. [file 12913_2025_12238_MOESM1_ESM.docx]

# Supplemental files

Supplement table 1 Number of codes and themes per domain facilitator and barriers and the inclusion in the themes and phenomena.

| **CFIR domain facilitator/barrier (joint code)** | | **Number of (#)** | | **Included in (yes/no)** |  |
| --- | --- | --- | --- | --- | --- |
|  |  | Codes | Themes | Overarching theme* | Phenomena^†^ |
| Innovation | Facilitators | 47 + 29 | 6 |  | 1,3 |
|  | Barriers | 41 | 8 | A, B, C | 2,3,5,6 |
| Outer Setting | Facilitators | 58 | 21 | A, C | 1,3,5 |
|  | Barriers | 55 | 31 | A, B, C | 2,3,5,6 |
| Inner Setting | Facilitators | 45 | 12 | A | 1,2,3,4 |
|  | Barriers | 25 | 8 | A, B | 1,2,3,6 |
| Individual (patient) | Facilitators | 11 | 5 | A, B | n.a. |
|  | Barriers | 6 | 3 | A, B, C | n.a. |
| Individual  (psychiatrist) | Facilitators | 17 | 6 | A, B | 1,3,5 |
|  | Barriers | 19 | 9 | A, C | 3,5,6 |
| Individual  (OH phycisian) | Facilitators | 18 | 9 | A | 1,2,4 |
|  | Barriers | 18 | 14 | A, C | 2,3,4,5 |
| Implementation Process | Facilitators | 52 | 16 | A | 1,3,5 |
|  | Barriers | 48 | 7 | B, C | 1,2,3,5,6 |
| *Overarching themes: A. The discussion of the scope and boundaries of the cooperation included in the innovation: What was the intervention?; B. The discussion of the smaller size of the target group than expected in the beginning: What was the target group?; C. Stigma associated with mental health problems or its absence.  †Phenomena: 1. Developers and healthcare providers shared belief in the significance of the problem and in the positive effect of the innovation.; 2. The need for clear roles and responsibilities of all involved in the treatment and rehabilitation process of workers with mental health problems – not only for OHS and psychiatrist.; 3. A need to anticipate and focus on the right timing and functionality of all the elements of the innovation components.; 4. Ambivalence towards OHS competencies and resources in supporting the RTW of workers with mental health problems. | | | | | |

Supplement table 2 List of CFIR domain codes, final themes, and phenomena

| **CFIR domain code (joint code)** and final themes | **Phenomena*** |
| --- | --- |
| **I. INNOVATION DOMAIN barriers (N=4)** |  |
| Patient related factors challenged the model's unwritten assumptions about the target group: Patient's varying treatment paths, issues related to stigma, and the fact that there were actually very little patients in this pilot in specialized psychiatric care. | 2,5,6 |
| Supporting return to work and continuing psychiatric treatment at the same time demands proactive and circumstantial collaboration and capabilities to understand the mechanisms of this twofold process. | 2 |
| The design of the model was based on the idea of using e-referral practice in different areas, but the ICT-infrastructures varies between wellbeing services counties and that created a considerable challenge to the implementation of the model. | 3 |
| The focus of the model did not include all cooperation paths and partners needed to answer the challenges related to mental health and RTW, but only focused on the referral path from PS to OHS. | 2 |
| **I. INNOVATION DOMAIN facilitators (N=3)** |  |
| For the pilot phase, critical deliverers were recognised and invited, and they were committed to the implementation. | 1 |
| The model included concrete practical and easy to use tools for professionals such as a digital referral system and a list of indications for referral. | 3 |
| The model is based on the main topics and themes from previous development work and existing service structures. | 3 |
| **II. OUTER SETTING DOMAIN barriers (N=12)** |  |
| Actors of the social insurance system were not informed of the pilot. | 2 |
| Cooperation structures differ from the implemented innovation. The consultation structure varies in the field of mental health locally and regionally. | 2 |
| Few or no cooperative patients: The size of the cooperation target group is know neither in psychiatric or OHS. | 6 |
| Individual level monitoring of patients treatment and rehabilitation paths does not work | 6,3 |
| RTW is thought to be impossible because of personal sickness related issues or workplace related discriminating attitudes or possibilities. | 5 |
| Self-stigma related to mental health problems. | 5 |
| Shortage of healthcare personnel. | 3 |
| Slow ICT-integration process: e-referral was not in use for everyone. | 3 |
| The innovation is not included in the usual care & rehabilitation recommendation and guidelines for mental health patients (care protocols). | 6 |
| The possibilities of OHS to support the RTW are not recognized and partly used poorly. | 2 |
| Unfortunate timing: The implementation of the wellbeing services counties at the same time weakened the commitment to the development work of the innovation. | 3 |
| Not enough time for joint development meetings between the innovation developers and deliverers (both PS and OHS). | 3 |
| **II. OUTER SETTING facilitators (N=8)** |  |
| [UpToDate] contact information of patients OHS provider in the patient data repository. | 3 |
| At workplaces accepting attitudes, opportunities, and supervisor's readiness to modify job according to one's needs for RTW. | 3 |
| Managers are committed to the implementation of the innovation because they see a potential benefit for their own organization. | 1 |
| Mid-level leaders in public health care and at workplaces are key implementation facilitators. | 1,3,5 |
| The e-referral practice or other working cooperation method in use. | 3 |
| The existing practice for patients' consent to data transfer. | 3 |
| There is a readiness for closer cooperation because collaboration is strongly believed to have significant positive societal and individual effects on work disability, prolonging working careers, and the quality of life of people with mental disorders. | 1 |
| There was an earlier development method implemented regionally to start with, tested in practice in somatic diseases, with public health care and occupational health care providers. | 3 |
| **III. INNER SETTING barriers (N=5)** |  |
| Direct contact persons were unclear between psychiatrists and occupational health services. | 2 |
| Personal to get on top of all tasks in the psychiatric care setting are missing. | 3 |
| Some interviewees of the healthcare providers were not actively involved in the implementation process. | 1 |
| The number of patients eligible for referral is unclear for all stakeholders involved, including the developer of the intervention | 6 |
| Timely ICT services to build the e-referral were missing and that caused some frustration with the development process. | 3 |
| **III. INNER SETTING facilitators (N=9))** |  |
| Clear management of tasks and responsibilities within the OHS and psychiatric care teams regarding developing and implementing the innovation. | 2 |
| Collaboration between OHS and psychiatric care services is easier in smaller than bigger areas (e.g. smaller municipalities) | 3 |
| Cooperation between OHS and psychiatric care providers was already existing, on which the innovation could build on and intensify. | 3 |
| Developers were satisfied with the project development | 3 |
| E-referral from psychiatric to occupational health care is already working for some healthcare providers and was perceived as a realistic possibility for every healthcare provider in the future. | 3 |
| Healthcare providers believe in the positive impact of the innovation on patients (health outcomes) and the healthcare system (resource use such as time and money) | 1 |
| Healthcare providers supported the aim and content of the innovation. | 1 |
| Healthcare providers are motivated and actively involved in the planning and implementation phase of the innovation. | 1 |
| Psychiatrists value and understand the role and expertise of the OHS for mental health patients | 4 |
| **OHS, Individual barriers (N=11)** |  |
| Continuous staff turnover and lack of physicians. | 3 |
| Development work is not counted as working time. | 3 |
| Digital medical records are not available for OHS. | 3 |
| Huge increase in OHS's workload because of mental health issues. | 4 |
| It is difficult to get private OHS providers, which are competing with each other, to operate regionally in a mutually agreed upon manner. | 3 |
| It is not easy in all jobs and workplaces to match the job to a person’s work ability need. | 5 |
| Local and regional actors of mental health care and OHS providers do not have comprehensive list of contact information of each other. | 2,3 |
| Occupational physicians and psychiatrists had different expectations for defining sick leave and guidance for this innovation was missing. | 3 |
| OHS need to increase their competence in dealing with employees with mental health problems. | 4 |
| The progress of the development work depends on the individual person, if the key person leaves the project, that endangers the implementation. | 3 |
| Frustration with the delayed integration of several patient data systems (related to e-referral). | 3 |
| **OHS, Individual facilitators (N=6)** |  |
| OHS can focus [only] on the RTW-process and co-operation with an employer. | 4 |
| A fulltime coordinator for cooperation with OHS in the service of a wellbeing service county. | Hypothetical |
| Among professional of [private] OHS a lot of readiness to cooperate. | 1 |
| OHS providers' chief physicians can be strong links in the operating model. | Hypothetical |
| Promising OHS's practices for following-up the success of one's RTW. | 4 |
| The development project gave enthusiasm and new regional professional networks. | 2 |
| **Patient, Individual barriers (N=3)** |  |
| Depending on the patients' situation, the return-to-work planning is not relevant or possible during the earlier stages of the recovery. | n.a. |
| The patient declines cooperation due to lack of confidence in stakeholders or because of symptoms of illness. | n.a. |
| The stigma associated with mental health problems hampers to seek treatment. | n.a. |
| **Patient, individual facilitators (N=2)** |  |
| A smooth operating model for patients. | n.a. |
| Expectations of outcome that improve individually patients' quality of life and support their recovery identity. | n.a. |
| **PS, Individual barriers (N=5)** |  |
| A special RTW model for people with mental health problems can in itself be stigmatizing. | 5 |
| Few or no patients suitable for this cooperation under the care of psychiatrists. | 6 |
| It was not easy to keep the indications of e-referrals to OHS in mind. | 6 |
| Psychiatrists' patients need help in coordination of their treatment and RTW. | 6 |
| The intense workload of psychiatrists. | 3 |
| **PS, Individual facilitators (N=3)** |  |
| Psychiatrists can pass their work[load] concerning the statements of work disability to OH-physicians. | 1 |
| Psychiatrists' patients are usually willing to cooperate. | 5 |
| The e-referral is easy to use for a psychiatrist. | 3 |
| **V. Implementation process barriers (N=5)** |  |
| Ambiguity about roles, resources, and the nature of the cooperation needed to implement the innovation. | 2 |
| Overall ambiguity about the size of the target group. | 6 |
| Some key actors did not understand work ability issues in the same way as it was needed for the innovation. | 1 |
| Stigma related challenges, such as work community's negative attitudes. | 5 |
| The e-referral usage that was included on the model as a ongoing process actually takes time to get up and running. | 3 |
| **V. Implementation process facilitators (N=6)** |  |
| A supportive and accepting workplace is crucial for the succes of the intervention. | 5 |
| Healthcare providers collect and discuss information about the success of the innovation on RTW related outcomes and the increase in cooperation between the healthcare providers. | 1 |
| Implementation steps of the innovation were defined in advance and the deliverers fit the implementation process to the context, such as using paper referral. | 3 |
| In some cases, the e-referral was already tested, implemented, and in use by some healthcare providers. | 3 |
| Innovation deliverers intentionally coordinate and collaborate on interdependent tasks to implement the innovation. | 1 |
| The developers of the innovation were reputable, credible, and trustable. | 1 |
| *Phenomena: 1. Developers and healthcare providers shared belief in the significance of the problem and in the positive effect of the innovation.; 2. The need for clear roles and responsibilities of all involved in the treatment and rehabilitation process of workers with mental health problems – not only for OHS and psychiatrist.; 3. A need to anticipate and focus on the right timing and functionality of all the elements of the innovation components.; 4. Ambivalence towards OHS competencies and resources in supporting the RTW of workers with mental health problems  Abbreviations:  CFIR: consolidated framework for implementation research  ICT: Information and communication technology  PS: psychiatrists  RTW: return to work  OHS: Occupational health services | |

Supplement 3: Interview guideline and themes from each perspective

**Interview guideline**

The data will be collected through six small-group interviews conducted via online meetings. The first interview will involve developers working on the project. The project has been initiated in two regions. The next four interviews will be reserved for representatives of psychiatric care and occupational health services operating within the networks of these regions. The sixth interview is reserved for potential collaborators with whom cooperation has been discussed but has not yet commenced.

Interviews will be conducted using a grounded theory-informed approach. After each interview, researchers will review the discussion. If a relevant topic related to the research questions emerges that is not covered by the initial interview themes, the interviewers will consider whether to include this theme in subsequent interviews. Additionally, the necessity of conducting the sixth interview will be assessed after the first five interviews. It is important to note that grounded theory itself will not be applied.

In the beginning of the interviews the interviewees read the Research Information Sheet that the interviewees had also received via e-mail before the interview. Interviewees are asked to give their consent to participate into the study and their answers are recorded.

According to Quality Implementation Framework, implementation process can be divided into four phases: 1) Initial Considerations Regarding the Host Setting 2) Creating a Structure for Implementation 3) Ongoing Structure Once Implementation Begins 4) Improving Future Applications. This framework should be shown to the interviewees in interviews via Teams to give understanding as to which phase they are currently working, and to understand the different phases. This applies only to the perspective of the developers.

**Interview themes from each perspective**

**Representatives of the developers of the model**

1. What key observations can you provide regarding the implementation of the new referral model?
2. What should have been considered during the planning phase?

- A diagram is presented based on research considerations for background material and to focus the discussion: interviewers introduce the Quality Implementation Framework picture.

1. How has the implementation of the model considered the differences between orthopedic surgery patients and psychiatric specialized care processes? Particularly from the perspective of occupational health support?

- Interviewers present the visualization of the model’s process to focus the discussion.

1. The contents of occupational health service agreements vary, and activities related to supporting work ability are still developing in many ways.

- Being aware of this, how realistic do you see the spread and embedding of the new referral model?
- Developers have played an important role as initiators of collaboration. What are your thoughts on this role after the project ends?

**Representatives of occupational health**

1. Do you think the preparatory work for the implementation of the new referral model was sufficient in all respects?
2. What factors or themes can you identify that facilitate the implementation of the new model?
3. What factors or themes can you identify that hinder the implementation of the new model?
4. What do you specifically expect from the new collaboration model?

- Are you planning to adopt the new approach?
- Have you received sufficient support in transitioning to the new approach?

1. What development suggestions do you have regarding the implementation of the new model?
2. The contents of occupational health service agreements vary, and activities related to supporting work ability are still developing in many aspects within occupational health services and collaboration. How feasible do you consider the utilization of the new model in supporting the return to work of patients who have been diagnosed with mental health issues?

**Representatives of psychiatric care**

1. Do you think the preparatory work for the implementation of the new referral model was sufficient in all respects?
2. What factors or themes can you identify that facilitate the implementation of the new model?
3. What factors or themes can you identify that hinder the implementation of the new model?
4. What do you personally expect from the new referral model?
5. What development suggestions do you have regarding the implementation of the new model?
